# Redefining the Diagnostic Approach to Adrenal Insufficiency: Re-Assessment of Baseline and Cortisol Increment Cut-Offs with the 1 µg Synacthen Test

**DOI:** 10.3390/medicina61071303

**Published:** 2025-07-19

**Authors:** Taieb Ach, Rim Dhaffar, Asma Ammar, Aycha Ghachem, Imen Halloul, Wiem Saafi, Hamza El Fekih, Ghada Saad, Yosra Hasni, Monia Zaouali

**Affiliations:** 1 Department of Endocrinology, University Hospital of Farhat Hached, Sousse 4031, Tunisia; dhaffarrim@gmail.com (R.D.); ghachemaycha@gmail.com (A.G.); imen.halloul22@gmail.com (I.H.); wiem.saafi@gmail.com (W.S.); elfekihamza@gmail.com (H.E.F.); ghada.saad6587@gmail.com (G.S.); y.hasni@gmail.com (Y.H.); zaoualimonia@yahoo.fr (M.Z.); 2 Faculty of Medicine of Sousse, University of Sousse, Sousse 4000, Tunisia; asmamedcom@gmail.com; 3 Laboratory of Exercise Physiology and Pathophysiology, L.R.19ES09, Sousse 4054, Tunisia; 4 Department of Hospital Hygiene, University Hospital of Farhat Hached, Sousse 4031, Tunisia

**Keywords:** cortisol, adrenal gland, adrenal insufficiency, synacthen test, ACTH, stimulation

## Abstract

*Background and Objectives:* Adrenal insufficiency (AI) is an endocrine disorder characterized by inadequate cortisol production, leading to non-specific symptoms that delay diagnosis. The Low Dose Synacthen Test (LDST) is commonly used to evaluate adrenal function, but traditional cortisol cut-offs may not accurately reflect adrenal function in all patients. This study aims to identify baseline cortisol cut-offs to accurately rule in and out AI, reassess the value of cortisol increment during LDST, and evaluate the accuracy of 30 and 60 min cortisol measurements in diagnosing AI. *Materials and Methods:* We conducted a cross-sectional analysis of patients who underwent LDST at Farhat Hached University Hospital. Diagnostic accuracy of baseline cortisol levels and cortisol increment was assessed using ROC curve analysis to determine optimal cut-offs for predicting LDST outcomes. *Results:* Among 163 patients (mean age 42.9 years, 63% female), baseline cortisol ≤ 5.35 μg/dL had 100% specificity but 41.5% sensitivity for LDST failure. Conversely, baseline cortisol ≥ 12.4 μg/dL had 100% sensitivity with 45.9% specificity. Single measurements at 30 and 60 min correctly classified 92.64% and 93.87% of cases, respectively. ROC analysis of 30 and 60 min cortisol increments showed high diagnostic accuracy (AUC 0.923 and 0.914, respectively). The optimal cortisol increment cut-off was 6.35 μg/dL for ruling in AI (99% specificity). *Conclusions:* We propose a novel AI diagnostic algorithm based on a single 30 min cortisol measurement, complemented by revised baseline cortisol cut-offs and cortisol increment as additional criteria. This approach may enhance diagnostic accuracy and minimize unnecessary testing, warranting further clinical validation.

## 1. Introduction

Adrenal insufficiency (AI) is an endocrine disorder characterized by an absolute or relative deficiency of adrenal cortisol production. Symptoms of AI are non-specific, leading often to diagnostic delays and misdiagnosis [1]. The epidemiological landscape of primary adrenal insufficiency (PAI) has evolved, affecting approximately 10–20 individuals per 100,000 in the population. In contrast, secondary adrenal insufficiency (SAI), is more common, with an estimated prevalence of 150–280 cases per million [2,3].

Timely identification of AI is critical as an acute adrenal crisis can be life-threatening [4]. Proper management involves well-established replacement strategies, yet diagnostic tests for assessing the hypothalamic–pituitary–adrenal (HPA) axis are suboptimal, and to a certain extent, clinical judgment is still vital to establish the need for replacement therapy [4]. The insulin tolerance test (ITT) is the reference test for confirming or excluding AI in adults and older children [1], but it has several limitations, including the requirement for a specialized hospital setting, and contraindications in certain patients, such as those with seizures, coronary artery disease, a history of stroke or in advanced age [5]. Other studies have suggested that the glucagon stimulation test (GST) may offer a safer alternative for assessing the HPA axis, particularly in patients where ITT is contraindicated or not feasible [6].

The cosyntropin stimulation test (CST), or ACTH (adrenocorticotropic hormone) test, is a safer method for assessing the HPA axis; it has a comparable sensitivity and specificity to the ITT and is widely used in outpatient clinics [2]. The CST test can be performed using either a high or low dose of cosyntropin. The high-dose standard short test (HDST) is the gold standard for diagnosing PAI, while the low-dose synacthen test (LDST) is considered in assessing SAI or tertiary AI (TAI) [7]. The LDST follows a similar procedure to the HDST and uses the same diagnostic thresholds [8], but the HDST may be unreliable in contexts where ACTH deficiency is partial, such as in patients using inhaled or topical glucocorticoids or after pituitary surgery [9].

Although there is no consensus on the timing of cortisol sampling, studies assessing the diagnostic accuracy of LDST usually rely on a peak cortisol level of ≥18 μg/dL regardless of whether this was taken at 30 or 60 min [10]. Additionally, pre-test cortisol values for predicting test outcomes vary across studies, likely due to the diversity of patient cohorts, assay methodologies, and sample sizes [11,12]. Reported lower cortisol cut-off values range from 3 μg/dL to 6 μg/dL, while upper morning cortisol levels range from 8.55 μg/dL to 18 μg/dL [11,13].

Prior research has sought to establish standards to include other CST-related parameters in predicting outcomes [10,14,15,16,17], with some reports suggesting that cortisol increment in AI diagnosis may offer additional information compared to baseline cortisol alone.

Our objective is to analyze LDST results from our health department to establish baseline cortisol cut-offs tailored to our patient population for ruling in and out AI. Additionally, we aim to test the reliability of either 30 or 60 min cortisol alone during LDST as a sufficient diagnostic test for AI and to re-evaluate cortisol increment cut-offs in outpatients to assess their diagnostic accuracy.

## 2. Materials and Methods

We conducted a cross-sectional study from 2015 to 2024 for all patients who underwent LDST at the endocrine day unit of Farhat Hached University Hospital, our designated institution. The study included patients presenting with clinical signs suggestive of PAI or SAI, provided they met the following criteria: age ≥ 18 years, absence of contraindications to LDST, including no history of allergies or intercurrent infections, and providing written informed consent. Patients were excluded from participation if they had chronic renal, hepatic, or heart failure; active neoplasia at the time of testing; incomplete cortisol measurements; pregnancy; use of combined oral contraceptive pills; or recent or current corticosteroid use within one month before the test. Additionally, those in the early postoperative period (less than six months) after pituitary surgery or recent exposure to radiotherapy (within six months) were also excluded. Patients were further excluded if they discontinued the LDST or if their clinical or hormonal data were insufficient for analysis.

### 2.1. Collected Data

Each patient was scheduled for an appointment to undergo the LDST, with data collected using a pre-established form. We collected information on age, gender, prolonged corticosteroid therapy, and personal and family medical history. Screening included signs suggestive of AI, body mass index (BMI), orthostatic hypotension, and melanoderma.

Metabolic exploration findings such as for hemoglobin, sodium, potassium, creatinine, calcium, and total cholesterol levels were also recorded. Hormonal treatment, especially corticosteroids and estrogen-progestin, were noted, with any hydrocortisone treatment discontinued at least 24 h before testing. Blood samples for cortisol measurement were collected in dry tubes. Due to the inconsistent availability of ACTH levels, we excluded this parameter from our analysis and did not stratify patients with abnormal LDST results into PAI or SAI groups. This exclusion aligns with our study’s objectives and does not detract from the overall study aim.

### 2.2. Test Protocol and Interpretation

The LDST was performed between 8 and 9 AM after fasting since midnight. ACTH was diluted in 0.9% sodium chloride, with the final dilution performed immediately before the test. A 1 mL dose was injected via a venous catheter in the forearm, with blood samples taken for cortisol measurement at 0, 30, and 60 min. The test was conducted by experienced medical staff, with patients informed of the procedures and objectives beforehand. All LDSTs were performed at a single institution over a 9-year period, using consistent protocols and the same assay platform. This methodological consistency reduces potential variability and enhances the internal validity of the study.

A normal response was defined as a stimulated cortisol level ≥ 18 μ/dL (497 nmol/L) at either 30 or 60 min. An abnormal response was <18 μ/dL at both time points.

Patients were categorized into two groups based on LDST outcome: Group 1 (G1), with suboptimal cortisol responses (<18 µg/dL at both 30 and 60 min), and Group 2 (G2), with adequate responses (≥18 µg/dL at either time point). This classification enabled the evaluation of clinical and biochemical predictors of adrenal insufficiency and the diagnostic value of baseline cortisol and cortisol increment.

Cortisol increment was defined as the difference between the baseline cortisol level (T0) and the cortisol level measured at 30 or 60 min following ACTH administration (T30 or T60).

Serum cortisol levels were measured using a Beckman Coulter radioimmunoassay (RIA) kit, with a sensitivity of 0.7 μg/dL and intra-assay and inter-assay coefficients of variation of 2.8% and 5.3%, respectively.

### 2.3. Statistical Analyses

All statistical analyses were performed using SPSS version 25.0. Comparisons between subgroups were made using Student’s *t*-test for numerical variables and Chi-squared tests for qualitative variables. Pearson’s correlation coefficient was employed to assess the relationship between two quantitative variables. The sensitivity and specificity of LDST in diagnosing AI were evaluated using receiver operating characteristic (ROC) curve analysis at various cut-off points. The optimal lower cut-off was determined to maximize the specificity and positive predictive value for identifying abnormal LDSTs, while the upper cut-off was chosen to maximize the sensitivity and negative predictive value for identifying normal LDSTs. A significance level of *p* < 0.05 (two-sided) was applied to all tests.

## 3. Results

### 3.1. Patients

The LDST was performed in 163 subjects, 60 male (36%). The mean age of the subjects was 42.9 years, range (18–85 years). Personal medical histories were comparable across groups, with high blood pressure being the most frequent condition in both G1 (18.5%) and G2 (16.3%) (Table 1).

Among the documented biochemical features suggestive of AI, hypoglycemia emerged as the most frequent finding leading to LDST, present in 32.5% of cases. Long-term corticosteroid therapy was a significant consideration, accounting for 65% of cases (Table 2).

### 3.2. Analysis of the Corticotrope Axis During LDST

**Figure 1:**
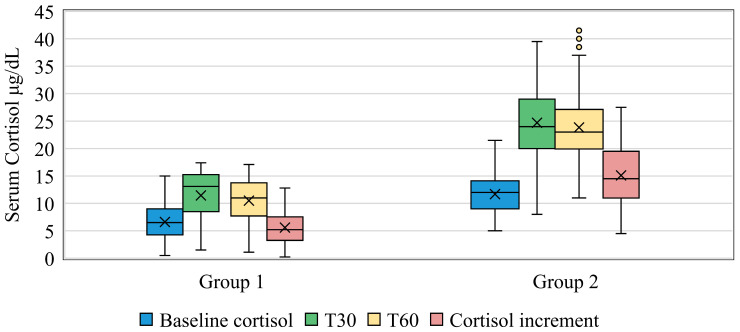
Comparison of serum cortisol levels between groups 1 and 2.

The baseline cortisol levels (T0) across the entire study population had a mean of 8.85 ± 3.86 µg/dL, ranging from 0.5 to 16.5 µg/dL. G1 exhibited significantly lower baseline cortisol levels (5.26 ± 2.14 µg/dL) compared to G2 (11.17 ± 2.73 µg/dL), *p* < 10^−3^ (Figure 1).

Following ACTH administration, the mean peak cortisol level was 20.47 ± 9.14 µg/dL, with most patients (58.3%) reaching this peak at 30 min (T30). G1 had a mean peak cortisol level of 10.53 ± 4.18 µg/dL at T30, while G2 had a higher peak of 24.68 ± 5.9 µg/dL (*p* < 10^−3^). Cortisol levels at 60 min (T60) slightly decreased in both groups but remained significantly higher in G2 (23.95 ± 6.01 µg/dL) compared to G1 (10.03 ± 3.92 µg/dL), *p* < 10^−3^ (Figure 1, Table 3).

The mean cortisol increment across the study was 11.62 ± 6.51 µg/dL, with G1 showing significantly lower increments (6.22 ± 3.25 µg/dL) compared to G2 (15.1 ± 5.6 µg/dL, *p* < 10^−3^). This trend was consistent at both T30 and T60, with G1 consistently displaying lower cortisol increments (*p* < 10^−3^) (Figure 1).

### 3.3. Baseline Cortisol and LDST Outcome Analysis

**Figure 2:**
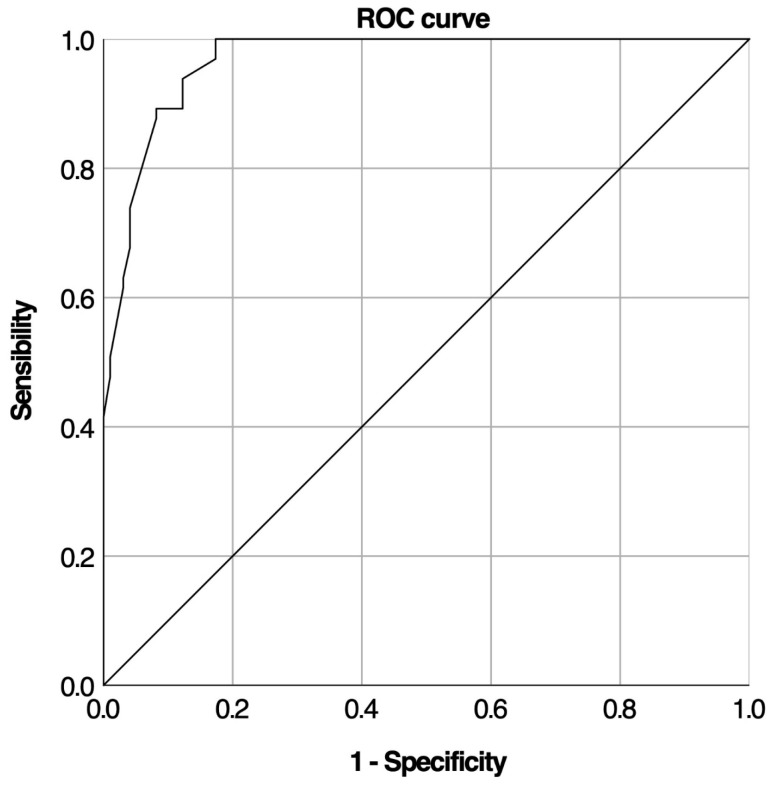
ROC curve for baseline cortisol levels during LDST.

A ROC curve analysis was conducted to assess the baseline cortisol test’s ability to predict LDST outcomes (Figure 2).

The test showed high accuracy with an AUC of 0.970 (95% CI: 0.948–0.992, *p* < 10^−3^). Analysis of 163 tests determined optimal cortisol cut-off values. The lower cut-off was set at 5.35 µg/dL, offering 100% specificity and 100% positive predictive value. The optimal upper cut-off value was set at 12.4, with 100% sensitivity and 100% negative predictive value (Table 4).

### 3.4. 30 and 60 Min Cortisol Measurements and LDST

**Figure 3:**
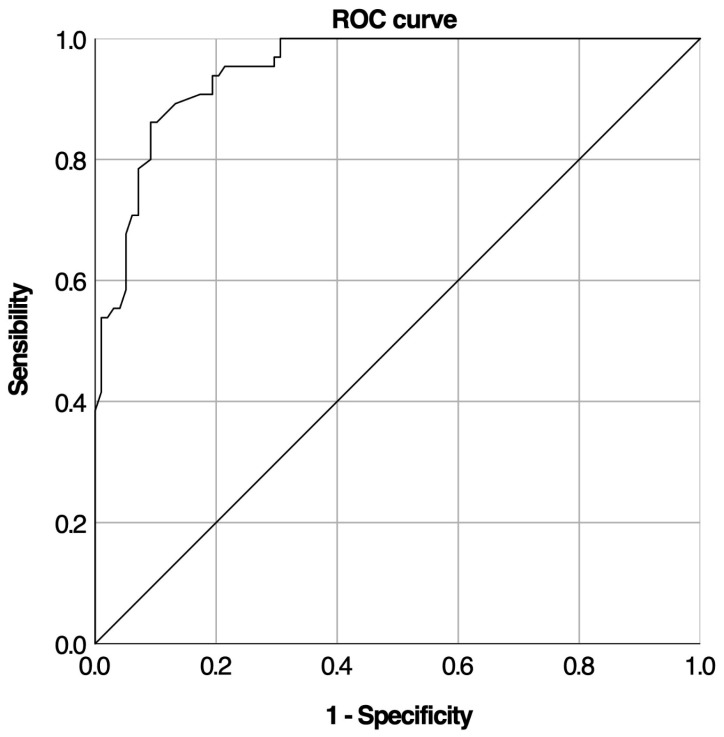
ROC curve for cortisol increment levels during LDST.

Among 163 subjects, 98 had a normal LDST result. Of these, 76 showed normal responses at both 30 and 60 min. A total of 12 subjects only peaked at ≥18 μg/dL at 60 min, having failed at 30 min, while 10 peaked at 30 min but dropped below 18 μg/dL at 60 min (Figure 3).

### 3.5. Cortisol Increment and Predictive Performance for AI Diagnosis

Logistic regression analysis revealed a significant inverse relationship between cortisol increment and LDST results; as the cortisol increment increased, the likelihood of an abnormal LDST outcome significantly decreased (*p* < 10^−3^) (Table 4). The ROC curve for cortisol increment demonstrated high diagnostic accuracy (Figure 3), with an AUC of 0.949 (95% CI: 0.919–0.979, *p* < 10^−3^).

The lower cut-off for cortisol increment to rule in AI which gave the highest specificity (99%) was 6.35 μg/dL, while the upper cut-off to rule out AI which gave the highest sensitivity (100%) was 11.95 μg/dL. These proposed upper and lower cut-off levels resulted in one false positive (FP) and no false negatives (FN), respectively. Other suggested cut-off levels also gave high sensitivity and specificity, around 90% (Table 4).

## 4. Discussion

Broadly, there are two groups of patients for whom it is crucial to evaluate adrenal function. The first group includes individuals who exhibit indicative symptoms (asthenia, weight loss, hypoglycemia). The second group consists of individuals who are at risk of developing AI, including patients who have undergone long-term glucocorticoid therapy, cranial irradiation, hypothalamic-pituitary disease, or pituitary surgery. Within our cohort, nearly 60% of our patients with suggestive symptoms had normal results to LDST, showing how non-specific most of the symptoms are.

Herein, we assessed the predictive value of baseline cortisol levels in diagnosing AI during LDST. ROC analysis identified that a morning cortisol level of ≤5.35 μg/dL confirms AI with 100% specificity, while a level of ≥12.4 μg/dL excludes AI with 100% sensitivity. Nearly half of the patients fell within these ranges, potentially eliminating the need for formal ACTH testing. For cortisol levels between 5.35–12.4 μg/dL, no conclusion can be drawn regarding adrenal functioning. By applying these cut-off values, we could reduce unnecessary LDSTs by 45% in our sample. However, only a small number of patients in this study had morning cortisol measurements taken before the test. The lack of routine morning cortisol measurements raises concerns, especially considering the cost of the test, emphasizing the importance of thorough medical documentation.

Our findings align closely with those of Perton et al., who proposed an upper morning cortisol cut-off of 13.6 μg/dL and a lower cut-off of 5.25 μg/dL [18]. However, it is important to note that the assay methods used in our study differ from those in Perton’s, as various assays for measuring serum cortisol can significantly impact interpretation [19,20]. Traditionally, a peak cortisol level of 18 μg/dL at 30 or 60 min post-ACTH administration is considered sufficient for normal adrenal function, though this cut-off can vary depending on the assay used. For example, newer monoclonal immunoassays, like the Elecsys Cortisol Generation II and Beckman Access Cortisol, typically produce cortisol values that are 20–30% lower than older polyclonal assays [21,22].

Finally, the potential need for glucocorticoid supplementation should not be dismissed when basal cortisol is normal, particularly if clinical symptoms strongly indicate AI. Ultimately, no cut-off value can replace good clinical judgment.

Numerous studies have measured cortisol levels at 0, 30, and 60 min after synthetic ACTH administration during HDST or LDST, consistently showing that cortisol peaks at 30 min [23,24,25]. Consequently, some experts recommend using the 30 min cortisol value as the primary indicator for assessing adrenal function [26,27], and certain centers have adopted this approach, limiting blood sampling to 0 and 30 min post-ACTH tests. However, many institutions, including ours, continue to assess cortisol levels at 0, 30, and 60 min and opt for a 60 min measurement in cases where synacthen is scarce due to financial constraints for patients. Anecdotal observations by endocrinologists at our institution have raised concerns about potential overdiagnosis of AI when relying solely on the 30 min cortisol level. In some patients, peak cortisol levels occur at 60 min, which could lead to a misclassification if only the 30 min measurement is considered. In light of this concern, we reviewed data in this study group and found that 22 patients would have been misclassified if we had relied solely on one-time sampling, with 10 and 12 patients at risk of overdiagnosis based on the 60 min and 30 min measurements, respectively.

Consistent with these findings, Cartaya et al. demonstrated the importance of measuring cortisol at both 30 and 60 min post-ACTH administration to avoid over diagnosing AI. They found that 9% of patients who failed at 30 min passed at 60 min, and 7% who passed at 30 min failed at 60 min [28]. The 60 min cortisol test remains valuable for ruling out AI, a practice supported by Chitale et al., who observed that patients with a “delayed response” often pass only at 60 min but have normally functioning adrenal glands [29]. Without the 60 min measurement, these patients might be unnecessarily prescribed long-term steroid therapy. However, different studies have shown conflicting results. Kumar et al. found that 5% of patients could be misdiagnosed if the 60 min sample is excluded, suggesting that a single 60 min measurement may be sufficient for diagnosing AI [30]. Similarly, Imran et al. reported that 9.5% of patients showed a suboptimal response at 30 min but reached the threshold by 60 min [31]. These studies and similar ones suggest that the 60 min value may be more reliable than the 30 min value [32,33]. Given our findings, our study supports the need to evaluate cortisol levels at both 30 and 60 min after LDST to prevent the overdiagnosis of AI.

Furthermore, our study underscores the significant finding that cortisol increment following LDST can accurately predict AI. For healthcare practitioners, relying on the 30 min cortisol measurement offers advantages such as convenience, reduced invasiveness, and time efficiency. The rationale behind using cortisol increment after ACTH stimulation stems from earlier studies, which suggested that relying solely on a single 30 min cortisol measurement could result in a higher rate of false positives for AI. We hypothesized that incorporating cortisol increment levels might improve diagnostic accuracy. Although the use of cortisol increment in diagnosing AI has been explored in several studies, the findings have been inconsistent. For instance, Struja et al. reported that delta cortisol does not provide additional diagnostic value compared to baseline cortisol alone, noting that its accuracy was inferior to basal cortisol [34]. Cortisol increment has also been proposed for use in critically ill patients [35]. Using serum delta cortisol after HDST in critically ill patients with a cut-off level of <9 μg/dL after peak serum cortisol can help diagnose AI [36]. However, Kadiyala et al. found no significant correlation between cortisol increment and basal cortisol, concluding that the incremental value was a poor predictor of HDST in acute medical admissions [37]. In the present study, most of the patients reached peak cortisol at 30 min, which agrees with a study by Cartaya et al. [28]. However, relying solely on the 30 min measurement raises concerns about AI overdiagnosis in approximately 7% of patients. To address this, we proposed incorporating a 30 min serum cortisol increment cut-off of 6.35 μg/dL as an additional diagnostic criterion. Using this proposed cut-off would have correctly identified all patients in the over-diagnosed group, except one, as not having AI. A diagnostic tool with high specificity is crucial to minimize false positives, and the lower cut-off of 6.35 μg/dL was found to offer the highest specificity with the fewest false positives. Several studies have recommended 30 min cortisol values as the preferred measure for evaluating AI in the LDST [23,31,38,39]. Based on these findings, we recommend performing only the 30 min cortisol measurement and calculating delta cortisol levels. If the 30 min serum cortisol level is less than 18 μg/dL, but the increment is at least 6.35 μg/dL, this is likely to indicate normal adrenal function.

Our proposed diagnostic approach for AI using the LDST aims to enhance the accuracy of diagnosis by addressing potential overdiagnosis associated when relying solely on 30 min cortisol measurements.

**Figure 4:**
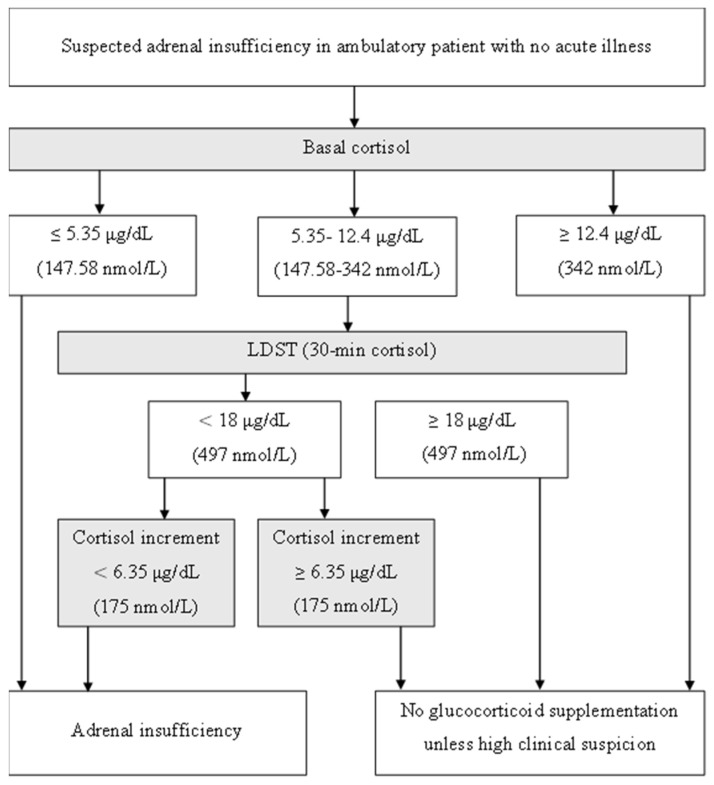
Diagnostic approach for suspected adrenal insufficiency using single time measurement (30 min sampling) during LDST.

Following LDST, if the 30 min cortisol level is ≤18 µg/dL, the cortisol increment should be assessed. A cortisol increment < 6.35 µg/dL suggests AI, whereas an increment ≥ 6.35 µg/dL likely excludes the diagnosis, except in cases of high clinical suspicion (Figure 4).

To the best of our knowledge, this is the first study from our geographical area that investigates cortisol increment as a diagnostic criterion for AI. This pioneering research offers a comprehensive analysis, with a diverse sample size that reflects the general outpatient population in terms of age, gender, medical history, and test indications. Our study is distinguished by an exhaustive analysis of LDST dynamic responses, including multiple ROC analyses. Moreover, this study introduces innovative approaches that could reshape clinical practice, particularly in addressing the shortage and high cost of synacthen. Importantly, all LDSTs were conducted in an outpatient setting, thus potentially avoiding the misinterpretation of cortisol that may result from the stress of being an in-patient. Unlike many other studies that have evaluated baseline cortisol levels without consideration of the time of day, our research focused on early morning cortisol levels (measured between 8–9 am).

We acknowledge some limitations in this study. First, we used retrospective data from a single center, and therefore, a prospective study would help further confirm these findings. Secondly, it is important to interpret this data considering the general limitations of LDST compared to ITT. Thus, we acknowledge the lack of confirmatory testing with gold standard tests or long-term longitudinal data to evaluate the accuracy of the diagnosis. It would be highly beneficial to complement these findings with further investigation into patient progression, as it would enable us to establish a “clinical pass” alongside a “biological pass”.

Although cortisol increment is a derived variable, it may offer additional diagnostic nuance, especially in cases where a single measurement yields an equivocal result. While our study did not include direct comparisons with ITT or HDST, our proposed increment threshold (6.35 µg/dL) aligns with thresholds reported in the literature for excluding AI in similar testing contexts. Future prospective studies comparing LDST increment criteria to gold standard tests like ITT are warranted to confirm the external validity of this approach.

## 5. Conclusions

AI is a potentially life-threatening condition with diverse manifestations, making its diagnosis challenging. Considerable controversy exists about which test is best, especially regarding LDST, which is considered an expensive test and entails significant inconvenience for the patient. A single measurement of baseline cortisol could significantly reduce the need for dynamic testing in AI diagnosis. Our findings suggest that both 30 min and 60 min cortisol measurements are valuable, but we also sought an additional criterion for cases where only one measurement is feasible, whether due to practitioner preference or financial constraints, a common issue in our institution. The 30 min cortisol increment from the LDST could also facilitate AI diagnosis. Due to variations between assays, exact cut-offs may vary. A larger prospective study is required before any definitive conclusions can be drawn.

## Figures and Tables

**Table 1:** Demographic, Clinical, and Biological Characteristics of Patients in Groups 1 and 2.

Variable	G1 (n = 65)	G2 (n = 98)	*p*-Value
**Average Age (years ± SD)**	43.26 ± 15.95	42.82 ± 17.18	0.358
**Gender**			
Males	23 (35.4%)	37 (37.8%)	0.642
Females	42 (64.6%)	61 (62.2%)	0.759
**Personal History**			
High blood pressure	12 (18.5%)	16 (16.3)	0.314
Dysthyroidism	9 (13.8)	9 (9.2)	0.352
Cardiovascular disease	2 (3.1%)	4 (4.1%)	0.214
Pituitary axis defect	4 (6.2)	2 (2)	0.172
Type 1 diabetes	4 (6.2)	8 (8.2)	0.631
Consanguinity	0	1 (1)	0.414
Celiac disease	0	2 (2)	0.247
Vitiligo	0	0	-
**BMI (kg/m^2^ ± SD)**	26.34 (±4.41)	25.63 (±4.96)	0.241
**Abnormal BMI n (%)**	34 (52.3)	49 (50)	0.773
Underweight	0	3 (3.1)	0.276
Overweight	20 (30.8)	34 (34.7)	0.602
Obesity I	9 (13.8)	4 (4.1)	**0.036**
Obesity II	4 (6.2)	5 (5.1)	0.516
Obesity III	0	3 (3.1)	0.276
**Orthostatic hypotension n (%)**	4 (6.2)	0	**0.013**
**Melanoderma n (%)**	10 (15.4)	1 (1)	**<0.001**
**Laboratory test abnormalities n (%)**			
Hyponatremia	13 (20)	11 (11.2)	0.250
Hyperkalemia	7 (10.8)	4 (4.1)	0.117
Calcium disorders	5 (7.7)	4 (4.1)	0.323
Anemia	17 (26.2)	23 (23.5)	0.697

G1: group 1; G2: group 2; SD: standard deviation; BMI: body mass index.

**Table 2:** Indicative signs and laboratory findings that prompt LDST in Groups 1 and 2.

	G1 (n = 65)	G2 (n = 98)	*p*-Value
**Functional signs**			
Asthenia	39 (60%)	55 (56.1%)	0.372
Anorexia	6 (9.2%)	11 (11.2%)	0.448
Fasting intolerance	2 (3.1%)	15 (15.3%)	**0.012**
Nausea and vomiting	1 (1.5%)	1 (1%)	0.640
Constipation	1 (1.5%)	1 (1%)	0.640
Diarrhea	0 (0%)	1 (1%)	0.601
Salt craving	0 (0%)	0 (0%)	-
**Physical signs**			
Weight loss	15 (23.1%)	28 (28.6)	0.276
Melanoderma	10 (15.4%)	1 (1%)	**<0.001**
Orthostatic hypotension	4 (6.2%)	0 (0%)	**0.013**
**Laboratory findings**			
Hypoglycemia	23 (35.4%)	30 (30.6%)	0.320
Hyperkalemia	0 (0%)	1 (1%)	0.601
Hyponatremia	0 (0%)	0 (0%)	-
**Clinical conditions**			
Long-term corticosteroid therapy	25 (38.5%)	26 (26.5%)	0.076
Corticosteroids withdrawal	4 (6.2%)	3 (3.1%)	0.340
Pituitary adenoma	12 (18.5%)	19 (19.4%)	0.526
Adrenal disease *	12 (18.5%)	5 (5.1%)	**0.006**
Post-pituitary radiotherapy	6 (9.2%)	11 (11.2%)	0.448
Post-pituitary surgery	8 (12.3%)	7 (7.1%)	0.199

* Adrenal disease: Unilateral adrenal incidentaloma, post-adrenal surgery; G1: group 1; G2: group 2.

**Table 3:** Distribution of cortisol levels and frequency of peak responses at different sampling times during LDST in study groups.

		T0	T30	T60
**Mean cortisol levels (µg/dL)**	**G1**	5.26 ± 2.14	10.53 ± 4.18	10.03 ± 3.92
	**G2**	11.17 ± 2.73	24.68 ± 5.9	23.95 ± 6.01
	***p***	**<0.001**	**<0.001**	**<0.001**
**Frequency of cortisol peak n (%)**	**G1**	NA	40 (61.5)	25 (38.5)
	**G2**	NA	55 (56.1)	43 (43.9)

**Table 4:** Diagnostic accuracy of baseline and cortisol increment cut-off values during LDST using ROC analysis.

Cut-Off	Spec%	Sens%	Sens-(1-Spec)	TP	TN	FP	FN
**Baseline Cortisol**							
**5.35**	100	41.5	0.415	27	98	0	38
**12.4**	45.9	100	0.459	65	45	53	0
**Cortisol Increment**							
**6.35**	99	53.8	0.528	35	97	1	30
**11.95**	69.4	100	0.694	65	68	30	0
**10.55**	78.6	95.4	0.74	62	77	21	3

Spec = specificity; Sens = sensitivity; TP = true positive; TN = true negative; FP = false positive; FN = false negative.

## Data Availability

Data are available if requested from the authors.
